# Animal research, ethical boundary‐work, and the geographies of veterinary expertise

**DOI:** 10.1111/tran.12594

**Published:** 2022-12-16

**Authors:** Alistair Anderson, Pru Hobson‐West

**Affiliations:** ^1^ School of Sociology and Social Policy University of Nottingham Nottingham UK; ^2^ School of Veterinary Medicine and Science University of Nottingham Nottingham UK

**Keywords:** care, ethics, interviews, laboratory animals, United Kingdom, veterinary expertise

## Abstract

The veterinary profession has been relatively understudied in social science, though recent work has highlighted the geographic dimensions of veterinary expertise. This paper draws on in‐depth qualitative interviews with Named Veterinary Surgeons (NVSs) working in UK animal research to demonstrate how and why they distinguish between ethical aspects of veterinary work in the spaces of the laboratory and general clinical practice. The paper mobilises the sociological concept of ethical boundary‐work to help understand how animal research – often assumed to represent a contentious ethical space – is constructed positively as a space for veterinary work. Findings suggest first, that NVSs differentiate between laboratory veterinary‐work and clinical work based on the scale at which veterinary expertise functions in the provision of healthcare to animals. Second, NVSs highlight a geography of veterinary authority in which veterinary expertise is felt to be more successfully applied in the laboratory compared with the clinic, where professional expertise competes with other sources of information and clients' finances and behaviours. Third, NVSs articulate a geography of consistency in which veterinary care in the laboratory is claimed to be more consistent between animals, as opposed to in the clinic, where animal experience may be influenced by individual owner characteristics. Overall, we show how through engaging in this kind of ethical boundary‐work NVSs are not only presenting a form of scientific practice as ‘ethical’, they are also constructing a professional topology of veterinary practice and expertise. Finally, the paper argues for greater attentiveness to veterinary geographies beyond the more routine spaces of veterinary practice.

## Short Abstract

This article mobilises the sociological concept of ethical boundary‐work to help understand how animal research – often assumed to represent a contentious ethical space – is constructed positively by Named Veterinary Surgeons as a space for veterinary work. We identify three aspects through which this boundary‐work is performed: an understanding of the scale at which veterinary expertise functions, a geography of veterinary authority in different spaces, and finally a consistency of veterinary care that is more applicable in the laboratory than the clinic. Overall we show how through engaging in this kind of ethical boundary‐work, NVSs are not only presenting a form of scientific practice as ‘ethical’, they are also constructing a professional topology of veterinary practice and expertise.

## 1 INTRODUCTION

Veterinary surgeons are ubiquitous in modern society. The veterinary profession, according to survey research conducted for the British Veterinary Association and Royal College of Veterinary Surgeons, is one of the most trusted professions in the UK (RCVS, 2019; VetFutures, 2015). Veterinarians hold some vital and visible roles, such as within agricultural processes and in the care of companion animals. However, veterinarians also serve a crucial and legally mandated function in the facilitation of animal research. This role has markedly lower public visibility within the ostensibly highly trusted veterinary profession.

Veterinary expertise is the focus of a nascent interdisciplinary social science literature examining the profession and its challenges, relations, and mobilities (e.g., Clarke & Knights, 2018; Cseke, 2023; Donald, 2019; Enticott, 2012, 2019; Hobson‐West & Jutel, 2020; Law, 2010; Morris, 2012). Geographers have made significant contributions to this area, for example in illuminating the situated expertise of veterinarians in bovine TB testing (Enticott, 2012), the international mobility of veterinarians following Brexit (Enticott, 2019), and ontological separations between humans and non‐human animals in veterinary ethics (Donald, 2019). Furthermore, Hobson‐West and Jutel's (2020, p. 6) reflections on the sociology of diagnosis highlights the occasionally porous borders between pet‐owners' decision‐making in human and animal healthcare. The veterinary profession, its expertise, and its sites of authority and care have a variety of fluid borders and require the navigation of complex professional borderlands This has implications for the understanding of fields such as employment, health, and non‐human animal geographies.

To date, minimal attention has been paid to how veterinarians themselves construct the topography of their profession and its professional niches. This is a potentially fruitful area of investigation for animal and health geographers illustrated not only by the ontological, economic, and practical challenges outlined above, but also by the varied ethical challenges present in different kinds of veterinary work. This paper therefore provides an example of ethical space‐making within the veterinary profession through examination of the ethical boundary‐work (Hobson‐West, 2012; Wainwright et al., 2006) performed by Named Veterinary Surgeons (NVSs) working in UK animal research laboratories. In choosing this approach, we assume that discourse is itself a form of action, worthy of detailed study. Our aim is thus not to report ‘opinions’ but, instead, to analyse and understand the precise discursive ways in which laboratory veterinary medicine and general veterinary practice are delineated, and identify why this matters.

## 2 ANIMAL RESEARCH AND THE ETHICAL SPACES OF SCIENCE

### 2.1 The role of NVSs

Using non‐human animals as stand‐ins for human bodies in research has a long history, and history of contestation (Franco, 2013). Whether and how such practices can be ethically justified has led to significant amounts of philosophic discussion, and more recent exploration in the social sciences. The latter has included, for example, comparison of the civic epistemologies that explain variations between countries (Davies, 2021); investigation of how imaginaries of animal and public sentience are built into regulation and laboratory practice (Hobson‐West & Davies, 2018); and examination of the ethical and regulatory challenges of the less common practice of conducting animal research in places outside the laboratory (Palmer et al., 2020). Indeed, the complex interweaving of human–animal healthcare relations, discursive ethical practices, and legal mandating discussed in this paper could also encourage future interaction between legal and animal geographies (see, for example, Neo & Ngiam, 2014).

Animal research in the UK is regulated via the Animals (Scientific Procedures) Act 1986 (herein ‘A(SP)A’). This creates a tripartite system, where the scientific establishment (the University or commercial organisation), the project (the particular plan of work), and the person carrying out research must be licensed. The involvement of veterinary professionals did not begin with the A(SP)A, however it did make the appointment of NVSs at all licensed establishments mandatory. The A(SP)A created statutory responsibilities for animal welfare, for which NVSs among other Named individuals such as Named Animal Care and Welfare Officers (NACWOs) are accountable (Hobson‐West & Davies, 2018). However, the role of the NVS is not as straightforward as the legislative mandate might initially suggest. This is because, as a member of the veterinary profession, NVSs also remain governed by their professional body, meaning they retain accountability to the establishment licence holder under the A(SP)A as well as having professional responsibilities to the animals under their care, the public, other veterinary surgeons, and the Royal College of Veterinary Surgeons under the Veterinary Surgeons Act (Ashall & Hobson‐West, 2018, p. 282).

Named Veterinary Surgeons are thus required to exercise professional judgement to reconcile potentially conflicting tensions arising from multiple professional accountabilities. An example might be as simple as the collection of a blood sample: if the procedure is for the animal's medical benefit then it falls under the Veterinary Surgeons Act and may be performed by the NVS. However, if the sample is for research purposes then the procedure falls under the A(SP)A and the NVS cannot carry out the procedure without the requisite personal licence. Such complex practical and regulatory navigation is not uncommon for veterinarians, as exemplified by Enticott's study of how veterinarians ‘manage the contingencies’ of bovine TB testing protocols, ‘patching together a scientific test with the needs of patients, colleagues and other material contingencies’ (2012, p. 79).

The NVS role harbours further complexities in that it is not simply a clinical role. Some commentators have argued that veterinarians are particularly well placed ‘to provide training, assessment, and supervision on what [are] considered to be veterinary interventions for scientific procedures’ (Poirier et al., 2015, p. 93). What this means is that NVSs are not only involved in the direct management of animal health and welfare, but are also involved in the training and education of researchers, ethical review, and the implementation of the 3Rs (the Refinement, Reduction, and Replacement of animals in research; Russel & Burch, 1959; Dennison & Petrie, 2020). The apparent ‘objects of care’ for veterinary expertise in the laboratory are consequently multiple, as they are in other sites of veterinary care such as on the farm or in the small animal practice (Hobson‐West & Jutel, 2020; Law, 2010; Law & Mol, 2011). However, as this paper will demonstrate, veterinarians working in animal research distinguish between the ‘objects of care’ in the laboratory and the ‘objects of care’ in general clinical practice using a particularly spatial form of ‘ethical boundary‐work’.

### 2.2 Ethical boundary‐work

In Gieryn's (1983) influential theory of boundary‐work, actors engage in discursive practice with the aim of separating what is a matter of science from what is not (for example, ethics or religion). Gieryn thus ‘sidesteps philosophical or epistemic debates to look at how scientists draw rhetorical boundaries between what is science and what is non‐science’ (Hobson‐West, 2012, p. 651). Crucially, the aim of this boundary‐work is to enable and maintain the socially privileged position of science, *vis‐à‐vis* other forms of knowledge and action.

However, in 2006, other scholars proposed an extension of the original concept. They claimed that ethics, rather than being constructed as separate from science, has now become an ‘integral part of maintaining the image of science’ (Wainwright et al., 2006, p. 735). In the case of controversial stem‐cell science, for example, Wainwright et al. (2006) argued that practices of *ethical* boundary‐work can be witnessed, whereby actors (including scientists) draw boundaries between what is ethically legitimate research activity and what is not.

Since its introduction, the concept of ethical boundary‐work has been applied to empirical research studies in a range of settings. Hobson‐West (2012) carried out interviews with senior laboratory scientists responsible for using animals in research. She argued that they perform ethical boundary‐work to portray their science as ethically legitimate, drawing a boundary between their own work and other methods such as human experimentation which they constructed as less legitimate. Likewise, Stephens (2013) interviewed scientists and animal activists involved in making and promoting in vitro meat, and showed how ethical boundary‐work was used to discursively underpin the promise of in vitro meat *vis‐à‐vis* animal liberatory narratives. In another example, Frith et al. (2011) studied infertility clinicians, illustrating how clinicians distinguished between ‘settled’ and ‘controversial’ areas of their work.

While the concept of ethical boundary‐work has been criticised for potentially reifying ethics (Hobson‐West, 2012), what unites these studies is the way in which they help to show ethical discourse as a matter of practice (Wainwright et al., 2006) – something that is done and achieved. In areas of controversial science then, the concept allows us to document ‘ethical complexity as it is understood by those involved in conducting the work’ (Stephens, 2013, p. 164). Using this approach is also helpful in identifying the people, places, or practices against which ‘science’ or ‘scientific research’ is defined. As previous research has shown, those working in sensitive areas have been seen to use a variety of rhetorical moves to delineate ‘ethical’ from disreputable ‘other’ practices (Hobson‐West, 2012; Michael & Birke, 1994; Stephens, 2013).

In this paper, we utilise the lens of ethical boundary‐work to help us make sense of interview conversations with NVSs. In doing so, we extend Wainwright and colleagues' previous work by demonstrating how, in the case of NVSs, ethical boundary‐work is distinctly spatial in nature in that veterinarians draw discursive boundaries between two physical professional spaces: the general practice clinic and the animal research laboratory. More specifically, the analysis reveals the way in which geography is brought into the enaction of ethical boundary‐work through three key mechanisms: conceptions of scale, the reach of authority, and consistency of welfare. Furthermore, it is argued that this ethical boundary‐work helps to distinguish between the multiple of veterinary cares (Law, 2010) required in these different spaces.

## 3 METHODS

This paper reports analysis from an interview study conducted as part of the Animal Research Nexus Programme (Animal Research Nexus Programme, 2019; Davies et al., 2020). The aim of this study was to understand the role of veterinarians as key and underexplored actors in the animal research laboratory. Ethical approval for data collection was granted by the School of Veterinary Medicine and Science at the University of Nottingham (approval number 1800160608), and data collection took place in 2018.

An interview guide was developed and discussed with an expert advisory panel of three NVSs, and was trialled during two pilot interviews with NVSs. The interview format and question focus were subject to revision as the data collection progressed. NVS participants were contacted through snowball sampling initiated via personal networks and a call out during a specialist conference. Thirty‐three NVSs were interviewed in person at locations chosen by the participants and all interviewees were currently working (full or part time) as an NVS. Interviews were transcribed by a third‐party under a confidentiality agreement. Transcripts were anonymised with all identifiable material regarding names, locations, and organisations removed. Such measures are arguably particularly important in an area of technoscience that remains contested in some quarters. Each transcript was assigned a random but gender‐specific pseudonym. These transcripts were analysed by the first author using NVivo 12.

The analytical approach adopted was reflexive Thematic Analysis (Braun et al., 2019), with the aim of inductively constructing analytic themes that reflect meaning‐based patterns across the interview dataset. The use of this inductive approach was particularly important as NVSs are an understudied group, and there was minimal prior research to draw on to inform the study and analysis. In the first cycle of coding, transcripts were coded line‐by‐line with an emphasis on prioritising the voice of the participant by focusing on their language and phasing through process and in vivo codes (Saldaña, 2016). This first round of coding was organised into broad categories with wide breadth of prospective content. Rather than simply summarising, the second round of more in‐depth coding developed these categories by identifying the patterns that underpinned the initial categories and creating analytical themes (Clarke & Braun, 2018). For example, the first round of coding identified the category of ‘Differentiating the NVS Role from General practice’. In the second round of coding, this material was grouped to include the way in which interviewees perceived and understood the dynamics of their profession, the place of ethics in different forms of veterinary care, and the nature of veterinarians' interactions with different stakeholder groups. However, this coding process was not linear, and in developing the second cycle of codes the first cycle categories were sometimes modified in title or content. Codes were also discussed between the authors. Overall, what is being described is a creative process; the themes did not ‘emerge from’ the data but were ‘active creations’ of the analyst (Clarke & Braun, 2018, p. 108). The findings therefore represent a kind of interpretive story developed from this data analysis and interacting with the biography of the researchers. In this paper, we use the overarching concept of ethical boundary‐work to organise these themes and understand how veterinarians create a positive ethical image of the laboratory as a site of professional veterinary work. Other project outputs use alternative approaches to focus on, and make sense of, different aspects of the interview data, including the career journey of the NVSs (Anderson & Hobson‐West, 2021).

The findings are presented in three sections that illustrate the veterinary geographies that form part of the ethical boundary‐work performed by the interviewed NVSs. First, NVSs differentiated between laboratory veterinary‐work and clinical work based on the *scale* at which veterinary expertise functions in the provision of healthcare to animals. Second, NVSs highlighted a geography of veterinary *authority* in which veterinary expertise is able to be more directly applied in the laboratory than the clinic, where professional expertise competes with other sources of information and clients' finances and behaviours. Third, NVSs articulated a geography of *consistency* in which veterinary care in the laboratory could be more consistent between animals, as opposed to care deliverable in the clinic which was constructed as more of a ‘lottery’, dependent on owners' characteristics.

## 4 CARING AT SCALE IN THE LABORATORY

The interview analysis suggests that veterinarians utilise concepts of care when explaining their role. However, what particularly struck us was the way in which the question of scale is made to matter. On the one hand, veterinarians argued that in the laboratory context their decisions may affect the design of housing facilities or the protocol of a study, and consequently the care of a large population of animals. For Maddison, one of the positives of the NVS role is ‘the difference we can make to not just one pet but 10,000 mice, [through] the little decisions that we can make’.

In the laboratory, the incorporation of veterinary expertise into the design of care (such as an animal's environment) and the legislatively defined role of veterinary advice in the laboratory tend to affect groups of animals at the same time and in the same place. This care at scale was contrasted with routine companion animal clinic work, where a veterinary surgeon tends to deal with individual animals and clients.

Nevertheless, the interview data analysis also revealed a challenging side of this scaling of care. Martin, for example, highlights the way in which the NVS role also has the potential to harm many animals or to cause harm that is unnecessary, if their protocols are incompatible with the research design:

You have this quarantine protocol […], or this vaccination protocol, worming protocol, is it good enough? Am I putting at risk the whole thing instead of one individual? And that brings some thinking and it's difficult to disconnect sometimes.

This distinction, comparing animal research with the more individualised approach of general practice, was also echoed by Macey:

The practice vet I think is more about healing the animal and making the animal feel good, and you don't worry that much about severity, because you just want to heal the disease. Then as an NVS, you are very worried about the project, the outcome of the project and what you can use, or you cannot use, the drugs.

Again, here is articulated a concern in general practice with healing the individual, contrasted with the NVS's worries about the project as a population of animals to be affected by veterinary decisions, and what care can or cannot be performed for the animals in the laboratory research context.

A final point links to the first extract from Maddison, about the capacity for the vet to care for multiple animals. Of course, when a veterinarian in clinical practice spays or neuters a cat, vaccinates a dog, or makes a referral for behaviour training, such interventions have implications for wider populations of animals beyond the individual patient. Nevertheless, our point here is that in the interview accounts, scale of care was used as a way to distinguish between clinical and laboratory practice.

To summarise, in this first section we have shown how NVSs use the idea of scale to distinguish between care in the laboratory and care in the clinic. Using the lens of ethical boundary‐work encourages us to consider precisely what is being achieved with this distinction. Our interpretation is that this is generally being done with the aim of presenting veterinary care in the laboratory in positive terms, and that in turn this presentation is deemed to be necessary within the wider social and political context in which the animal research laboratory represents a controversial space (Hobson‐West, 2010; Smith & Wolfensohn, 2006).

Having said this, the analysis also suggests that NVSs feel responsibility to care not only for the animal or animals, but are also required to care for the experiment or for the science itself. This affirms Druglitrø's contention that in the animal research laboratory, animals are ‘situated in a network of other elements that [need] to be cared for’ including the ‘quality and reliability of science’ (Druglitrø, 2018, p. 656). Our analysis suggests that NVSs, then, are involved in this complex form of caring, both ‘for animals as biological beings and scientific tools and caring for the scientific system’ (2018, p. 656).

While this finding is in line with Druglitrø, our points about scale are somewhat different from existing literature. For example, other authors have argued that the growing physical scale of the animal research facility can serve to inhibit the possibility of time spent with each animal, and thus the chance to innovate refinements in care (Greenhough & Roe, 2018, p. 694). Likewise, as Davies has reflected, ‘the potential for shared suffering fits uneasily within large commercial mouse houses (containing up to 60,000 animals), in which animal care is increasingly as routinised as the standardised animal housing’ (2012, p. 633). By contrast, our analysis suggests that NVSs interviewed in this study use ethical boundary‐work to positively contrast the more population‐level care possible in the laboratory, despite scientific infrastructural constraints, with the more individualistic, time‐pressured focus of general clinical practice. The concept of ethical boundary‐work has thus helped attune us to *how* NVSs describe their daily work and, crucially, also the *why*. Put simply, constructing the laboratory as a site of care helps them present their role as ethically justifiable and, in turn, helps maintain the ‘image of science’ (Wainwright et al., 2006, p. 735).

## 5 THE AUTHORITY OF VETERINARY EXPERTISE IN THE LABORATORY

### 5.1 The complex role of finance across veterinary spaces

In this section, we consider the way in which NVSs discuss the scope of their own particular form of professional expertise, given the particular role of finance in different veterinary spaces. In this study, the veterinarians recounted a number of examples in their career where their decision‐making ability was experienced as limited due to issues arising from other actors. Interestingly, however, these examples often related to clinical work. Nevertheless, the laboratory setting itself was not devoid of challenge.

The first limitation highlighted involved the financial burdens that exist in general practice. For Mack, for example, this meant that little investigative work was done, which led to a reliance on presumptive diagnoses and a lack of surveillance:

I worked in practice and […] you are [depending] on what the farmer wants to pay. You could never investigate anything, they wouldn't pay for it…

[…] In veterinary practice, for example, you've got an animal with a neurological condition or something and in the practice we would just say ‘this animal had a stroke’ or something. […] You don't know what it was and you just, you didn't have the methods either, to investigate, and you probably didn't have a lot of knowledge about neurology in the first place. And then you just say ‘it had a stroke’, and that was easily understandable to most of the clients because they know a stroke, how it looks in humans, and that was it. Then you cull the animal because of a stroke.

In Mack's laboratory work, however, understanding cause of death was considered important to potentially prevent other animals in the laboratory dying from similar causes: ‘Every single animal will be submitted for a post‐mortem examination […] because you want to know why something died.’

These extracts illustrate the complexity of veterinary power and authority in the clinic. In the first part, Mack is expressing some frustration with the role of animal‐owner finances in acting as barrier to further investigation. This sentiment was echoed by Nancy, who felt that ‘here [in the laboratory] I find that you're liberated of that cost constraint’. In the second part, Mack goes further to elaborate on the impact of these barriers, which was that he was only able to use a fairly commonplace and translatable diagnosis (stroke) without further investigation, which would not happen in his laboratory veterinary work (third part of the extract). The point is that Mack is recounting a story from practice in order to articulate the limits to his authority caused by a lack of resource. The ‘stroke’ diagnosis here is based on the veterinarian's expertise without confirmatory testing. This establishes an ethical boundary in his account between clinical work and laboratory work, where a full diagnosis would more usually be required to confirm the condition of the deceased animal in order to protect the broader laboratory animal population (Druglitrø, 2018).

However, the wider analysis suggests a more complicated story, where, in some cases, finances were also seen to matter inside some laboratories. For example, Molly described similarities between general practice and the NVS role in the context of financial decision‐making in the pharmaceutical industry:

I think [animal research] can be dolled up in doing the best for patients and bringing new medicines for patients, which is important, but at the end of the day, it's like being back in general practice. It's about making money. […]

I think it's sort of shareholders, they're calling the shots and … focus on the patient, that's one of our big straplines but if we're not making money, if we're not getting the drugs through, we're not going to survive. We've had ethical dilemmas of, we have to do this study here or we have to prepare these dogs with […] devices to do this study here because otherwise these people won't have any jobs. If we're seen to put too many obstacles in the way, then the work will go elsewhere and these people will lose their jobs and that's absolutely dreadful, but people have said that to me on more than one occasion.

Together these examples demonstrate some heterogeneity among NVSs regarding the perception of separation between veterinary roles in terms of the impact of financial limitations. While Mack and Nancy felt that these limitations predominantly existed in general practice, Molly clearly highlighted a context in which financial limitations also affected decision‐making in the laboratory with an impact on ethical outcomes.

The second limitation identified by interviewees was less about finances and more about epistemology, in the sense of who is considered to have authority to ‘know’ the animal. For example, some veterinarians talked in negative terms about the extent to which animal owners in the clinical setting rely on online health information. Oscar, for example, bemoaned the direction they felt that general practice was taking:

I don't particularly like the way that general practice is going, where with the internet everyone knows as much as you do: sometimes so it's hard when the owner of the animal is telling you what's wrong with the animal.

This unease was also expressed and expanded on by Mia, using sarcasm, who included breeders among the information sources favoured by some clients:

So, people will come in and they will have read lots of things, not necessarily things that are correct but they're much more willing to listen to someone … Well, they've always been willing to listen to the breeder. The breeders always know more than anybody else, don't they? But now they're more willing to listen to all sorts of forums and information that actually is not evidence‐based at all.

The examples highlighted in this section so far suggest the ways in which general practice was constructed as a noisy epistemological space, in which the voice of the veterinarian has to compete with, or take into account, a variety of other factors, including clients' time, financial contexts, and various other sources of information accessible to the public. This professional concern over a wavering authority or ability to act has also been found in previous studies focused on clinical veterinary practice (Hobson‐West & Jutel, 2020; Volk et al., 2011). To return to the accounts presented in the current study, this anxiety contrasts with the laboratory where cost constraints are argued to be less inhibitory to diagnoses or treatment, though the presence of financial requirements may still lead to challenges and tensions.

### 5.2 ‘What the NVS says, goes’: The enrolment of non‐veterinary actors

As previously noted, veterinarians are just one actor in the animal research laboratory. The data analysis revealed the way in which NVSs report enrolling other actors in order to achieve their professional aims. However, according to the interview accounts, the extent to which this was deemed necessary, and how successful this enrolment is, differed in NVS' accounts between general practice and the NVS role. For example, Michelle argued that:

The one thing I found which is a big advantage [in the laboratory] is that the vet has much more say about how the animals are looked after and can ensure the animals are looked after in the way you're advising than you do with owners in general practice. Because if you're the vet [in the laboratory] and you say the animal has to be looked after in this way and you want this done, you know that the people are going to do what you're telling them to do. You know that the animal technicians looking after them are very experienced and very good at handling animals, and you haven't got that confidence in owners to do things like that.

For Natasha, who shared these sentiments, there was a clear distinction to be made in terms of the scientific and anatomical knowledge of other actors that made the general practice and laboratory settings different when prescribing care for animals:

I think you deal with the same things just in different ways across all the fields, so in general practice, you still want good animal welfare but you're coming at it from the perspective of working with clients that don't necessarily know a lot about science and a lot about anatomy and a lot of research and that type of thing, whereas in this field you're working with people that are incredibly intelligent in science and it's just two different people that you have to explain things to in completely different ways.

This extract highlights the crucial role of interpersonal skills for veterinary surgeons. While there may not be pet owners as clients in the laboratory space, the veterinarian still needs to find ways to explain the required care to the relevant actors involved. However, the stakeholders present in each setting are used by the NVSs to erect another ethical boundary that constructs the laboratory as a site of expert care (a ‘big advantage’, in Michelle's words) in which they, as the veterinarian, have confidence that prescribed care will be competently delivered compared to, for example, in general practice with the pet‐owning public.

For Oliver, the two professional spaces also have different timescales in terms of urgency. In his case, he attributes this to the role of regulation. Regulation here buttresses the laboratory as a site of animal care (also see Wainwright et al., 2006), where the limitations that are claimed to exist in general practice are removed:

First of all the regulations are such that what the NVS says goes. There's no sort of, you don't get the same sort of kickback that you get from clients. When you say to people ‘can you come in on Tuesday?’ and they say ‘oh no, I can't, I'm busy, I'll come in on Thursday afternoon’, basically if you say to someone [in the laboratory] you need to come and look at your animals, they have to come in and look at their animals.

Other interviewed NVSs made similar distinctions. Parker, a former farm animal veterinarian, discussed how relying on what the technical staff tell him in the laboratory differs from his experience on the farm, where some clients may not follow through with the care he recommends:

I suppose at least with the technicians, if they tell me there's something wrong, there's likely to be something wrong [laughs]. You know what I mean? And also, if you ask for things to be done, you know it will be done rather than, you know, you can send people home with medication and it comes back a week later and it looks no better and then when you probe, you realise actually, the medication hasn't been given.

To be clear, we as analysts are not making the claim that veterinarians have more capacity to act in the laboratory than on the farm or in the small animal clinic. Rather, we are suggesting that NVSs themselves construct these spaces of authority differently. In general practice, there is a complex triad between the veterinarian, the owner, and the animal (Grimm et al., 2018). Our study suggests that animal research may also be seen as a triad, but between the veterinarian, the animal, and other colleagues perceived to be fellow experts in care.

Utilising the lens of ethical boundary‐work again attunes us to why this matters. While daily work in the laboratory space was not described as devoid of challenge, the NVSs broadly constructed the space of the laboratory as an expert site with more consistent execution of care practices due to the urgency associated with veterinary instructions and the perceived reliability of colleagues in overseeing or delivering veterinary‐directed care. The result is that the laboratory site is constructed as a relatively more care‐ful space less impacted by some of the challenges that come with the limitations of clients and finances in general practice. This is important in a context where animal research is sometimes presented as a less ethical space, or a space in which veterinarians should not even be (Animal Aid, 2016).

## 6 CONSISTENCY OF WELFARE IN THE LABORATORY

### 6.1 Burdens of complexity in veterinary treatment

In animal research, animals are usually required to be standardised in terms of characteristics and care (Druglitrø, 2018; Kirk, 2018), reflecting the sequestration of the laboratory space as a micro‐world of controlled conditions and ‘stagings of nature’ (Livingstone, 2003, p. 27). Outside in general practice, however, the veterinarian may encounter animals with a range of potential or perceived health variations. In the interviews, NVSs discussed this difference, articulating the view that in laboratory veterinary medicine the tendency was for animals coming to the attention of the NVSs to be healthy rather than sick. This was a perception present for both companion animal and farm animal care in the NVS accounts. For example, Mabel, whose laboratory work involved dogs, cats, and fish, recounted one of her earliest impressions of NVS work:

My experience of starting was initially, it was very different from general practice because 99% of the time the animals that you see are perfectly healthy and they don't have anything wrong. And you're seeing them because someone's noticed some minor thing, which may be a cause for concern: their feed intake has dropped today or they've been a bit off their hard food for three days, ‘so can you just have a look?’ Or, their behaviour's not quite right. So most of the time I was seeing things because someone thought there might be something wrong or because it's got a minor abrasion or a minor cut, and nobody else wanted to make the call about whether they can just ignore it or whether they need to treat it or not …

Participants' accounts varied on whether this difference in welfare was coincidental or a marker for systemic differences between the general practice and animal research settings. Martin, for example, who also worked with dogs, cats, and fish, felt that this may be due to ‘luck’:

So as NVSs, and again I can only talk from my experience, I've had the luck of mainly dealing with healthy animals, we don't usually have animals that are very ill or have very complicated cases. In some ways, that takes away the burden because if I was in practice, I'm sure I would be thinking about this case, how complicated it is, what can I do, blah blah blah, this is not common for us, at least for me as NVS.

A consequence of this apparent ‘lucky’ difference for Martin is a reduced burden of complexity compared with treating cases in general practice. For Oscar, however, involved in the care of large animals in the laboratory, the difference is perceived as systemic and is attributable to the difference in animals' financial value between the research and agricultural business context:

We give the animal disease but we look after them and they're not reared for a commercial environment. I charge for the bed and breakfast for the animals but it wouldn't be commercially viable to produce them for the value of the animal; it's only for the value of the research that I can charge as much as I can for the animals. And, because I charge a lot for the animals, they're well looked after, there's lots of staff to look after them. So, […] the animals here have a much greater value than animals on the farm and because of that, they're looked after much better.

Existing literature can help make sense of this material. For example, Clarke and Knights have argued that (non‐laboratory) veterinary work is ‘predominantly commercial, where the language of profit and growth tends to dominate’ with the effect of turning ‘animals and themselves, into productive beings’ (Clarke & Knights, 2022, p. 681). However, in Oscar's version of this narrative, laboratory animals are valued higher than they would be in farm animal practice, precisely because they are not directly being reared for a ‘commercial environment’. Rather, the high cost of their housing is explained due to the ‘value of the research’. Once again, the ethical boundary constructed in this narrative is between general practice and laboratory work. In general practice, part of a veterinarian's professional role may be to work with animal owners to negotiate between a ‘Cadillac’ versus a more affordable ‘Ford Pinto’ treatment plan (Morris, 2012, p. 175; and for a broader discussion of ways in which non‐humans come to bear capitalist value, see also Clarke & Knights, 2018, 2022; Collard & Dempsey, 2016; Hobson‐West & Jutel, 2020). By contrast, the interview analysis suggests a lower burden of professional complexity in the animal research laboratory, with the veterinarian claiming to assume greater control over the form that animal care takes.

### 6.2 The non‐negotiability of welfare when animals are data

For some of the NVSs in the sample, animal welfare was constructed as more tightly controlled in the laboratory setting than the clinic. As already described, the parameters in which decisions are made about animal welfare in general practice incorporate a number of variables that are explicitly removed in the animal research setting. For example, in veterinary clinical practice, some owners may prolong the life of a pet beyond that which the veterinarian would ideally choose. This scenario is claimed not to occur in the animal laboratory, due to the presence of specific controls such as humane end points, as elaborated by Nicholas:

It's the classic example of you've got the 80‐year‐old lady with a 17‐year‐old cat which has got kidney failure, you know it should be put down but you know it's probably her last companion and you send the cat home, even though it's the wrong thing to do, for the cat. Whereas here, it's an end point, you've got a predefined end point, you know what you're doing it for and so if an animal needs to undergo pain and distress, you've got a very clear purpose and it's for an answer and that answer is generally involved in the alleviation of a lot of human suffering, that's what the intent is here. So I find that a clearer decision.

This kind of practice–laboratory comparison is not new; the ‘inhumane treatment of pets’ has previously been contrasted to ‘the stringent policing of the treatment of animals in laboratories’ by animal research scientists (Michael & Birke, 1994, p. 198). Furthermore, the promissory claims (about alleviating human suffering), such as those present in this extract, have also been identified in previous work with senior laboratory scientists (Hobson‐West, 2012, p. 653; more broadly Wainwright et al., 2006). As Law (2010) reminds us in his analysis of the 2001 Foot and Mouth outbreak, there are multiple objects of care for veterinarians. In Law's (Law, 2010, p. 60) case there were four ‘objects of care’ as veterinarians cared for the animals but also the farmer, themselves, and the ‘bigger picture’. We would argue that this multiplicity of care is being replicated in our study. By referring to the example of an elderly lady in general practice, Nicolas alludes to care for the client; reference to alleviating human suffering in the laboratory alludes to the ‘bigger picture’. As will be highlighted below, these accounts are central to the ethical boundary‐work undertaken by Nicholas to frame laboratory veterinary‐work as a positive ethical comparator to the more commonly occupied (Robinson et al., 2019) general practice role.

The perception of tight control exercisable in the laboratory was echoed in multiple NVSs' narratives about animal welfare, with Nadir, for example, outlining a number of ‘non‐negotiable’ areas and graphically describing the life of a companion animal as a ‘lottery’ in comparison with that of a laboratory equivalent:

However, from the animal's point‐of‐view I really don't think they care how we feel about them. What they benefit from is what we do for them. In this environment the skill levels are non‐negotiable. The levels of attention are non‐negotiable. The environment, the amount of space the animals have, and whether or not they've got food or water, all potential areas of neglect, are non‐negotiable. So the day‐to‐day life of the animals is assured in a way that with companion animals it isn't.

I think a companion animal has a bit more of a lottery in their existence perhaps than these research animals. A cat can be lucky and have good owners, or a cat can be unlucky and have owners that treat them very badly. In this environment that doesn't happen. Certainly deliberate harms are carried out on animals in a way that is predetermined but you know where you are. So as a vet I sit in an environment where I have some level of certainty about what I'm dealing with, and I find that quite helpful.

Crucially, this level of predetermination and control was perceived to allow greater professional influence for veterinarians in ensuring welfare, as exemplified by Melody:

I think I would now really struggle with practice as far as the cruelty to animals because here [in the laboratory], we can stop it. In practice it's constant, people can't say goodbye or they don't have the money. The powerlessness because you have to give your client the options, they choose the best way but at the end of the day the power isn't yours.

However, our analysis suggests that it is not as simple as equating the laboratory with complete veterinary control. As highlighted by Natasha, they are also themselves subjects of control due to the requirements of the regulation and adherence to A(SP)A:

I still certainly do find myself having to stop and be like, ‘Wait, you can't just do this treatment because of …’ whatever's being done [in the study], so there is certain things that I still catch myself and it's not like general practice where I can just treat with whatever I want. Actually I have to stop and think, and also think about severity limits and the defined endpoint and things like that.

This account again alludes to a further object of care for the veterinarian in the laboratory – as already discussed above, the research study itself. Under A(SP)A, a veterinarian without a personal research licence may only perform procedures for the animal's benefit (as under the Veterinary Surgeons Act) and not for research purposes. Additionally, the project design may require, for example, that certain medications are not used. The NVS may therefore be unable to perform treatment for a particular animal, in order to care for the research study as an object.

As previously noted, Druglitrø has stressed the dual role of laboratory animals as ‘compound objects of care’ (2018, p. 656), both technological and biological, part of a scientific infrastructure and part of a biological species. This compound status was recognisable in some NVSs' narratives in relation to their place within scientific studies as just highlighted, but also in relation to the way the same biological species might be treated for a severe condition in practice and in the laboratory. For example, this was neatly articulated by Oliver, a part‐time NVS primarily working in general practice, who drew on the requirements of scientific infrastructure to contrast the possibilities for veterinary treatment of animals in clinic and laboratory:

If an animal's unwell the data it's providing may be unreliable or unrepresentative of a healthy animal. […] So if I see an animal, for example, that has a broken leg then really there's probably little point in doing any treatment, euthanasia's the best option. Now, of course, that can be at odds to the ethos that we have in practice, whereby pet animals we'll try and treat them unless they've got to a stage where suffering is obvious and we can't guarantee that they're going to improve … And that's one thing that I did find slightly odd when I started doing NVS work and I was treating the dogs at [name of organisation] and I hadn't really appreciated that I had to make sure that whatever treatment I prescribed for the dog wouldn't interfere with the scientific research …

In summary, interviewees generally constructed the laboratory as a space characterised by consistent and non‐negotiable levels of animal care. These accounts again construct ethical boundaries in relation to the consistency of delivery of veterinary care, here articulated in the context of animals' value as data. However, as the final extract has illustrated, this is more complex and nuanced in that some requirements of the laboratory may be ‘at odds’ with the animal care ethos of clinical practice. Oliver emphasises that animals in the research laboratory serve a specific purpose and their welfare is defined in relation to that purpose. This echoes Oscar's comments, cited above, regarding commercial animals and their welfare on a farm compared with in the laboratory – their value has been defined by their purpose and that value is intimately linked to the care they receive. In other words, it is the animals' role as research data which can create caveats on the care they actually receive. Similarly, that data suggest that while veterinarians in general practice may have a wider scope for care in theory, actual care is limited by the kind of role the animal is playing for the individual client or farmer.

The lens of ethical boundary‐work again helps us to understand not only how but why the idea of standardisation and consistency of animal welfare in laboratory and clinical veterinary contexts was used in the interviews to discursively construct ethical boundaries between these veterinary niches. As seen previously, regulation was part of this boundary‐work in defining ‘non‐negotiable’ areas of care in the laboratory. However, the analysis also reveals other ways in which the place of animals in laboratory and clinical settings underlaid ethical contrasts. As a start, the animals in the laboratory were claimed to have a higher economic value due to their place within scientific infrastructure than animals reared in commercial environments. While separation on the basis of an animal's ascribed value was less prominent regarding companion animals, a reversal of the veterinarian's clinical negotiating position was evident in accounts of laboratory work. Rather than the financial aspects of veterinary care undermining professional credibility due to the need to negotiate an animal's care, as has been described in the literature (e.g., Clarke & Knights, 2018; Morris, 2012), the ‘value of the research’ was framed as empowering for the NVSs and their ability to deliver the care they felt to be necessary.

However, laboratory animals' value as data underlaid other objects of care for NVSs (Law, 2010) – first care for the ‘big picture’ in promissory discourses of the alleviation of suffering through scientific progress (Hobson‐West, 2012; Wainwright et al., 2006) and second in care for the research studies themselves through the maintenance of standardisation (Druglitrø, 2018). The animal geography of this place‐linked valuing of animals thus introduced significant complexity arising from the varied access animals had to treatment in NVSs' accounts. On the one hand, NVSs perceived themselves has having greater control through the non‐negotiability associated with regulation, but on the other hand, some NVSs reported options were more limited in what could be offered to laboratory animals, given the importance of standardisation. Overall, this section suggests a complex picture of discursive ethical boundary‐work whereby the animal research laboratory is normally, but not entirely, constructed as the more ethical space. As throughout this paper, this ethical boundary‐work matters in the way it helps to justify and support the norms and practices of laboratory animal science.

## 7 DISCUSSION AND CONCLUSION

This paper has drawn on novel interview data to analyse the way in which UK NVSs discursively draw ethical boundaries between the space of the laboratory and the space of the clinic. By using the sociological concept of ethical boundary‐work, this is revealed as a kind of action. Ethical boundary‐work here draws attention to the detail of discursive strategies, and encourages a focus on the why, as well as the what, of claims‐making. In this study, the why is to create a positive image of the animal research laboratory as a site of professional veterinary work. This ethical boundary‐work is necessary, one could argue, given the social context of animal research, which is still regarded as controversial in some quarters (Smith & Wolfensohn, 2006), and the reported high level of public trust in the veterinary profession (RCVS, 2019; VetFutures, 2015).

While previous studies drawing on ethical boundary‐work refer to space, this has generally been in passing, or conceptual rather than literal. Wainwright et al., for example, refer to ethical work sequestered ‘from the space of the laboratory and placed with public regulatory bodies’ (Wainwright et al., 2006, p. 742), and both Frith et al. (2011) and Wainwright et al. (2006) refer to their participants' delineation of some version of a discursive ethical space to occupy. Hobson‐West goes further and notes that the scientists in her study construct a metaphorical ethical space that is ‘a safe discursive location’, as well as delineating between the physical separation of animal use inside and outside of the ‘hallowed space’ of the laboratory, such as the use of animals for food or the treatment of animals identified as pests (2012, p. 659). However, this current paper is novel in foregrounding the importance of geographic dimensions as tools by which ethical boundary‐work is achieved for what is a regulatorily centralised profession (Hobson‐West & Timmons, 2016). The NVSs in this study identified areas and characteristics of laboratory veterinary care that were both spatially and ethically distinct from general clinical practice.

First, care was focused on different scales in the NVSs' accounts of general practice and laboratory veterinary medicine. Many of the interviewees began their careers as small animal veterinarians and reported that their ability to deliver care was tempered by the complexity of individual cases and the financial and practical capabilities of different individual clients. In their narratives, this was clearly contrasted to the laboratory setting, where care was constructed as more standardised. Ethical boundary‐work here was perhaps partly used to validate their own personal career choice or trajectory (Anderson & Hobson‐West, 2021). However, this work is also directed more broadly to validating the very presence of veterinarians in the controversial space of the animal research laboratory. This is achieved via claims that in the laboratory their expertise has greater impact on a larger number of animals than would be routine in companion animal practice. Nevertheless, examples also reveal complexity in that individual treatment options are not always possible or may generate anxiety, given the overall experimental protocol.

Second, the analysis points to a geography of veterinary authority present in the NVSs' accounts. In the general practice setting, interviewees created an image of their voice as one among many. We did not conduct ethnographic observation and are unable to empirically evaluate this claim. However, this perception is arguably borne out in existing literature on the companion animal field which identifies the importance of online animal health information, and the perceived material impacts of ‘Dr Google’ on the relationships between pet‐owners and veterinarians (Hobson‐West & Jutel, 2020; Volk et al., 2011). Limits to veterinary expertise have also been noted in agriculture: For example, Buller et al. have identified the way in which rapid diagnostic tests may challenge the ‘unique authority’ (2020, p. 10) of veterinarians to perform diagnoses. By contrast, the continued authority of veterinarians within the laboratory setting was presented as high and prominent within a professionalised and formalised care context (Davies et al., 2018; Kirk, 2018). Once again, this ethical boundary‐work has a key purpose in helping to construct the laboratory as a space where veterinary expertise is positively impactful. The unique contribution of this paper is to illustrate the ways in which NVSs mobilise the spaces of their profession to discursively push back against opposition (for example, Animal Aid, 2016) to the involvement of their ‘trusted’ profession (RCVS, 2019) in animal research. Nevertheless, it is important to recognise that this ethical boundary‐working was not uncontested. Some of the analysis explored how dilemmas in the lab are reported as challenging, for example for the way in which finances also play a sometimes‐problematic role in how NVSs are able to perform their role.

Third, this paper shows how the NVSs' accounts drew discursive ethical boundaries between the welfare of animals seen in general veterinary practice and in the laboratory. In the laboratory, the value of the animals as data and the heavy regulation of the laboratory space was used to create an image of the laboratory as a space of consistent care. In such accounts, animals treated in other veterinary contexts like general practice may be subject to good care but also poor care depending on their ticket in the ‘lottery’ of pet‐owners. Conversely, the regulation of laboratory spaces and presence of other expert animal handlers was perceived as being generative of a more consistently positive space of veterinary care. As before, this ethical boundary‐work is a route through which to validate the laboratory as a space of (veterinary) care. This work is arguably another form of resistance to social movement campaigns which have criticised veterinary involvement in animal research and, more broadly, a form of resistance to the idea that laboratory veterinarians are ‘caught in the middle’ between scientists and antivivisectionists (Smith & Wolfensohn, 2006, p. 811). However, once again, this was not uncontested. In the third theme, NVSs also noted that they need to care for the science as well as the animals when making treatment decisions. The ethical boundary‐work performed by NVSs to push back on negative perceptions is therefore again qualified by the requirements of their role as a veterinarian for animal research, rather than simply as a clinical practitioner.

As well as focusing on an understudied actor in the laboratory, our paper was also motivated by wider ambitions. In short, we hope to encourage more empirical research on the laboratory or animal research site itself. Our observation is that while geographers routinely engage in researching the spaces where veterinary expertise is commonly evident, from the more‐than‐human family home (e.g., Fox, 2006; Power, 2008) to circulations of agricultural capital (e.g., Buller et al., 2020; Cseke, 2023; Enticott, 2012), the laboratory is a less often considered site for such research. For example, it may be that existing approaches in geography can be fruitfully applied to the laboratory. While the field of health geography has a longstanding interest in unequal access to (human) healthcare (Rosenberg, 2014), the accounts of veterinarians presented here imply that there is also an unequal access to animal healthcare, and that this access is partly determined by the roles – and spaces – ascribed to particular animals (Buller, 2016; Hovorka, 2019; Srinivasan, 2013). Additionally, there may be further overlaps between animal, health, and legal geographies in understanding ambiguities in the spatialisations of categories of veterinary expertise (Neo & Ngiam, 2014).

Finally, we also hope to encourage more empirical and theoretical consideration of the varied roles of veterinarians and the impact of veterinary expertise in contemporary society. More specifically, our contention that NVSs engage in ethical boundary‐work and that this has geographic dimensions may help open up potential new avenues for future research. After all, in engaging in this kind of ethical boundary‐work, NVSs are not only presenting laboratory animal research as ‘ethical’, but are also constructing a professional topology of veterinary practice – where domains are defined as more or less conducive to the successful application of veterinary expertise. Further work on the varied kinds of veterinary professional work would thus be useful. More specifically, we would advocate the use of methods such as comparative ethnography, which, rather than focusing on discourses of practitioners, could focus on the differences in how care work or ‘response‐able practices’ (Davies et al., 2018) are enacted by veterinarians across different spaces (Law, 2010), including the laboratory, the small animal clinic, the farm, or the slaughter‐house. Furthermore, a more global comparative perspective would also be welcomed as this paper has focused squarely on UK veterinarians. Geographers would be particularly well placed to analyse the extent to which our claims also apply internationally, and to highlight the ‘global flows’ (Enticott, 2021) of veterinary experts and veterinary expertise in and beyond animal research.

## Data Availability

The dataset on which this paper draws has been anonymised and deposited in the UK Data Archive, subject to an embargo: Davies, G., Greenhough, B., Hobson‐West, P, Kirk, R., and Roe, E. (2033) *Animal Research in the UK, 2017–2023*. UK Data Service. SN: 8942, DOI: 10.5255/UKDA‐SN‐8942‐1.
